# Temperature-Sensitive Sensors Modified with Poly(N-isopropylacrylamide): Enhancing Performance through Tailored Thermoresponsiveness

**DOI:** 10.3390/molecules29143327

**Published:** 2024-07-15

**Authors:** Lei Yang, Guangwei Qiu, Yuanyuan Sun, Luqiao Sun, Xiaoguang Fan, Qiuju Han, Zheng Li

**Affiliations:** 1 School of Petrochemical Engineering, Liaoning Petrochemical University, Fushun 113001, China; yanglei@lnpu.edu.cn (L.Y.); qiuguangwei@stu.lnpu.edu.cn (G.Q.); sunluqiao@stu.lnpu.edu.cn (L.S.); 2 College of Engineering, Shenyang Agricultural University, Shenyang 110866, China; 2022240009@stu.syau.edu.cn; 3 School of Environmental and Safety Engineering, Liaoning Petrochemical University, Fushun 113001, China; lizheng.zz@163.com

**Keywords:** poly(N-isopropylacrylamide), screen-printed platinum electrodes, temperature-sensitive sensors

## Abstract

The development of temperature-sensitive sensors upgraded by poly(N-isopropylacrylamide) (PNIPAM) represents a significant stride in enhancing performance and tailoring thermoresponsiveness. In this study, an array of temperature-responsive electrochemical sensors modified with different PNIPAM-based copolymer films were fabricated via a “coating and grafting” two-step film-forming technique on screen-printed platinum electrodes (SPPEs). Chemical composition, grafting density, equilibrium swelling, surface wettability, surface morphology, amperometric response, cyclic voltammograms, and other properties were evaluated for the modified SPPEs, successively. The modified SPPEs exhibited significant changes in their properties depending on the preparation concentrations, but all the resulting sensors showed excellent stability and repeatability. The modified sensors demonstrated favorable sensitivity to hydrogen peroxide and L-ascorbic acid. Furthermore, notable temperature-induced variations in electrical signals were observed as the electrodes were subjected to temperature fluctuations above and below the lower critical solution temperature (LCST). The ability to reversibly respond to temperature variations, coupled with the tunability of PNIPAM’s thermoresponsive properties, opens up new possibilities for the design of sensors that can adapt to changing environments and optimize their performance accordingly.

## 1. Introduction

In the ever-evolving field of sensor technology, the pursuit of enhanced sensitivity, selectivity, and reliability remains paramount. Among the various strategies employed to achieve these goals, the integration of stimuli-responsive polymers, such as poly(N-isopropylacrylamide) (PNIPAM), has garnered significant interest. PNIPAM, a water-soluble polymer exhibiting unique thermoresponsive behaviors, offers a promising route for modifying sensors to impart temperature sensitivity [1,2]. PNIPAM is renowned for its ability to undergo a reversible phase transition in aqueous environments, exhibiting a hydrophilic-to-hydrophobic transition around the lower critical solution temperature (LCST) of approximately 32 °C [3,4]. This transition is characterized by a conformational change in the polymer chain, leading to significant alterations in its physical properties. By harnessing this thermoresponsive nature, PNIPAM-modified sensors can be designed to detect and respond to temperature variations with high precision and sensitivity. The integration of PNIPAM into sensor surfaces provides a means to tailor their response characteristics. The hydrophobic-to-hydrophilic transition of PNIPAM modifies the surface properties of the sensor, altering its wettability, permeability, adsorption behavior, and interactions with the surrounding medium [5,6,7]. These changes, in turn, influence the signal transduction mechanisms of the sensor, enabling it to detect and transduce temperature signals into measurable outputs. Moreover, the tunability of PNIPAM’s thermoresponsive behavior offers further versatility in sensor design. By adjusting the molecular weight, concentration, or copolymerization degree of PNIPAM, the LCST and the magnitude of the phase transition can be precisely controlled [8,9]. This allows for the development of sensors with tailored sensitivity ranges and response profiles, optimized for specific applications and operating conditions.

Tailoring the thermoresponsiveness of sensors utilizing PNIPAM facilitates more precise and sensitive detection of temperature changes within or around the critical range. Depending on the specific application, the thermoresponsiveness of PNIPAM-based sensors can be tailored to respond to temperature changes within a customized temperature range. This ensures that the sensors are optimized for the specific environment or process being monitored. By tailoring their thermoresponsiveness, PNIPAM-based sensors can maintain stability and reliability across a broader range of temperatures. This is crucial for applications that require continuous monitoring under variable temperature conditions. The tailored thermoresponsiveness of PNIPAM-based sensors enables their use in a wide range of applications. For example, they can be used in biomedical applications for monitoring temperature changes in living tissues, in environmental monitoring for tracking temperature gradients, or in industrial processes for controlling operating temperatures. PNIPAM-based sensors with tailored thermoresponsiveness can be easily integrated with other materials and devices. This allows for the development of multifunctional sensors that can provide additional information, such as regarding electrical conductivity or mechanical strength, in addition to temperature sensing.

Various techniques, ranging from physical methods to chemical and biological approaches, can be employed to achieve an effective binding of PNIPAM with the sensor surface. Physical methods involve the utilization of non-covalent interactions, such as electrostatic attractions, hydrophobic interactions, and intermolecular forces, to affix PNIPAM onto sensor surfaces [10,11,12]. One such approach is the simple adsorption of PNIPAM molecules onto the surface through immersion or deposition techniques [13,14]. The adsorption process can be controlled by adjusting parameters like concentration, temperature, and pH to ensure optimal coverage and binding strength. This method offers simplicity and versatility, though the binding may not be as robust as with covalent bonds. On the other hand, chemical methods rely on the formation of covalent bonds between PNIPAM and reactive groups present on the sensor surface [15,16,17]. Surface functionalization is often employed to introduce suitable reactive moieties onto the sensor surface, which can then react with complementary groups on the PNIPAM chains [18]. Techniques like surface-initiated polymerization or grafting-to reactions allow for the controlled growth of PNIPAM chains directly on the sensor surface, providing a dense and uniform coating [1,19]. Chemical methods offer stronger and more durable attachments but may require more complex synthetic steps and careful control of reaction conditions. Moreover, biological methods have emerged as promising alternatives for the integration of PNIPAM with sensor surfaces. These methods harness specific biological interactions and recognition mechanisms to achieve high selectivity and affinity. For instance, biorecognition molecules such as antibodies or peptides can be immobilized on the sensor surface and then used to capture PNIPAM molecules bearing complementary biological ligands [20,21]. This approach offers high specificity and can be tailored to target specific PNIPAM conformations or functionalities. It is worth noting that the choice of the integration method depends on the nature of the sensor material, the desired PNIPAM coating properties, and the intended application of the sensor. For example, sensors made of metal oxides or polymers may lend themselves naturally to certain physical or chemical methods, while biosensors may benefit from the high specificity of biological methods. Additionally, the compatibility and stability of the PNIPAM coating under different operating conditions should also be considered.

In this study, with the aim of achieving effective attachment between PNIPAM and the electrode surface, while also ensuring the formation of a stable film, a two-step film-forming strategy named as “coating and grafting” developed by our team was employed [22,23]. Initially, siloxane bonds and hydroxyl groups were incorporated into the polymers. Subsequently, a uniform layer of PNIPAM copolymer solution was deposited onto the electrode surfaces, which possessed hydroxyl groups. By applying heating and annealing, the siloxane groups within the copolymer chains would bind with both intramolecular and intermolecular hydroxyl groups, as well as the hydroxyl groups originating from substrate surfaces. This process enabled PNIPAM copolymers to be grafted onto the electrode surface through a methanol removal reaction that occurred between siloxane bonds and hydroxyl groups.

In this study, NIPAM was combined with hydroxypropyl methacrylate (HPMA) and 3-(trimethoxysilyl) propyl methacrylate (TMSPMA) as supporting components to fabricate thermoresponsive coatings with a crosslinked structure. By employing a two-step film-forming approach on the surfaces of screen-printed platinum electrodes (SPPEs), a series of temperature-sensitive electrochemical sensors with different grafting densities were successfully developed. These sensors exhibited a remarkable sensitivity to hydrogen peroxide (H_2_O_2_) and L-ascorbic acid, indicating good film permeability. Additionally, notable temperature-dependent variations in the electrical signals were observed as the electrodes underwent modest temperature fluctuations above and below the LCST. Our report introduces a novel, straightforward, and practical approach for creating a stable PNIPAM copolymer film on platinum electrodes. Furthermore, it marks the first investigation into assessing the impact of graded surface wettability and variations in film thickness on sensor performance and functionality. The modification of sensors with PNIPAM offers a promising avenue for enhancing their temperature sensitivity and versatility. By harnessing the unique thermoresponsive behavior of PNIPAM, electrochemical sensors that are more responsive and tunable to temperature variations have been developed.

## 2. Results and Discussion

### 2.1. Preparation of P(NIPAM-co-TMSPMA-co-HPMA) Copolymer Film-Modified Platinum Working Electrode

**Figure 1:**
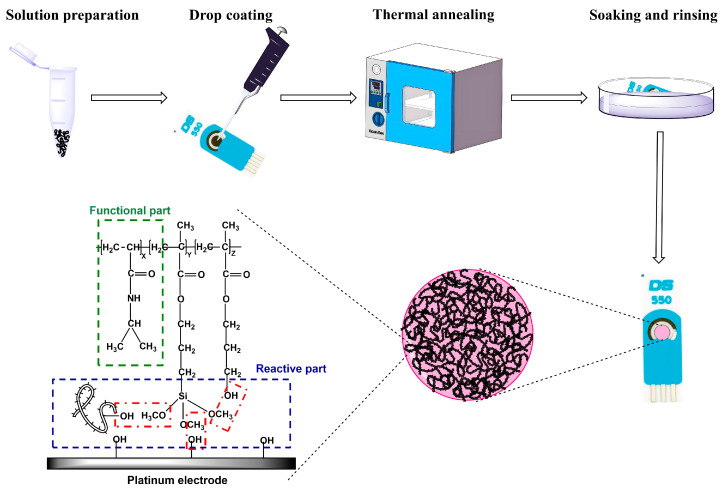
Preparation strategy of a P(NIPAM-co-TMSPMA-co-HPMA) copolymer film-modified platinum working electrode.

The platinum working electrodes of SPPEs were modified with P(N-isopropylacrylamide-co-3-trimethoxysilylpropyl methacrylate-co-hydroxypropyl methacrylate), P(NIPAM-co-TMSPMA-co-HPMA) copolymers. Therein, NIPAM served as a key reactant, endowing the product with temperature-responsive characteristics, and TMSPMA functioned as a cross-linker, remaining stable throughout the polymerization process while effectively forming crosslinks with hydroxyl groups of HPMA and substrate surfaces [22,23]. The condensation reaction between hydroxyl and methoxyl groups gave off methanol, while the newly created –Si–O– bonds facilitated integration within the film and bonding with the platinum surface containing residual hydroxyl groups, as shown in Figure 1.

The LCST of P(NIPAM-co-TMSPMA-co-HPMA) copolymers was determined to be approximately 25 °C, as obtained by a dynamic light scattering (DLS) technique using a Malvern Zetasizer (Nano ZS, Malvern Instruments Ltd., Malvern, UK). The weight-average molecular weight (M_W_) of the copolymers was about 72.6 kDa, and the number-average molecular weight (M_n_) was nearly 34.3 kDa, with a polydispersity index (M_W_/M_n_) of 2.117, as measured by gel permeation chromatography (GPC, Waters 515, Waters Corporation, Milford, MA, USA). The actual molar ratio of the copolymers was in agreement with the initial feed ratios of 20:1:1, as confirmed by ^1^H-NMR spectra obtained via a Bruker AV 400 NMR spectrometer (Bruker Corporation, Fallanden, Switzerland).

### 2.2. Characterization of P(NIPAM-co-TMSPMA-co-HPMA) Copolymer Film Modified Platinum Working Electrode

#### 2.2.1. Chemical Composition

**Figure 2:**
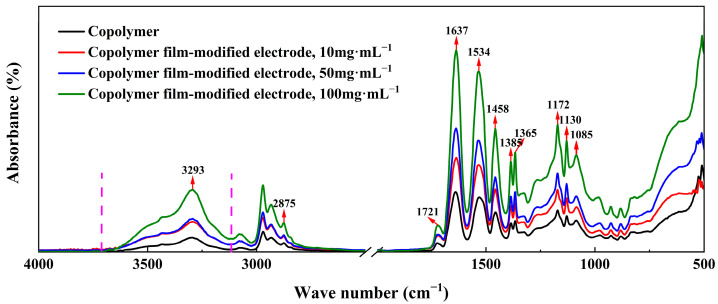
ATR-FTIR spectra of P(NIPAM-co-TMSPMA-co-HPMA) copolymer film-modified platinum working electrode.

ATR-FTIR analysis, as depicted in Figure 2, initially demonstrated the successful covalent attachment of P(NIPAM-co-TMSPMA-co-HPMA) copolymer films onto the platinum working electrodes of SPPEs. NIPAM was characterized by the presence of doublet absorption peaks corresponding to isopropyl groups, located at 1385 and 1365 cm^−1^, along with absorption peaks attributed to amide II at 1534 cm^−1^ [24,25]. Moreover, the successful incorporation of siloxane groups was evident from the absorption peaks observed at 2875 and 1085 cm^−1^ [26,27]. Additionally, the absorption peaks located at 1721 and 1172 cm^−1^ were attributed to ester groups originating from HPMA and TMSPMA [26,27]. For the copolymers composed of NIPAM, TMSPMA, and HPMA, the intense and broad absorption peaks observed within the frequency range of 3100 to 3700 cm^−1^ could be attributed to hydroxyl groups found in HPMA and amide bond II of NIPAM [27,28]. However, when it came to platinum electrodes modified with P(NIPAM-co-TMSPMA-co-HPMA) copolymer film, the absorption peaks in the 3100~3700 cm^−1^ region became sharper due to a condensation reaction that took place between hydroxyl and siloxane groups.

It can also be clearly observed from Figure 2 that both the intensity and the extent of the characteristic absorption peaks associated with the copolymers increased notably as the concentration of the preparation solution rose. This increase provided indirect evidence of an elevated quantity of the various constituent monomers within the copolymers.

#### 2.2.2. Grafting Density

The results showed that there was little change in the weight of bare SPPEs before and after heating, indicating no material loss or oxidation changes. To assess the grafting density of P(NIPAM-co-TMSPMA-co-HPMA) copolymers, a comparison was made between the masses of SPPEs prior to and following grafting. The grafting density results provided the approximate values of 2.53, 4.16 and 5.95 mg∙cm^−2^ for the SPPEs modified with P(NIPAM-co-TMSPMA-co-HPMA) copolymer films, prepared at the final concentrations of 10, 50, and 100 mg∙mL^−1^, respectively. It was noteworthy that there was a positive correlation between grafting density and coating concentration of copolymer solution. This supported the conclusion that an increased availability of molecular chains for crosslinking resulted in thicker copolymer films being formed within the allocated reaction time for the coating process [29,30].

Despite this, it was evident that the grafting density of the copolymers did not increase in a linear manner with respect to the concentration of the copolymer solution. This observation indicated that the copolymers gradually and uniformly covered the surface of the platinum electrode, ultimately completing the cross-linking process from a monolayer to a multilayer molecular chain structure. Prior to thermal annealing, an increase in the solution concentration resulted in the deposition of more copolymers on the surface of the platinum electrode. However, upon annealing, the presence of steric hindrance made covalent cross-linking between intramolecular and intermolecular chains more challenging. Consequently, the trend of film thickness growth slowed down at higher copolymer concentrations.

#### 2.2.3. Equilibrium Swelling

The study evaluated the equilibrium swelling properties of P(NIPAM-co-TMSPMA-co-HPMA) copolymer film coated on platinum electrodes at temperatures of 20 °C and 37 °C. Since the copolymer chains needed time to adopt an appropriate conformation at a given temperature, the modified electrodes were allowed sufficient response times to reach an equilibrium swelling state prior to measurements [31]. Table 1 further revealed that the copolymer films in all three cases exhibited a marked increase in water absorption at the temperature below the LCST, whereas they became dehydrated at higher temperature. As expected, the swelling ratio of P(NIPAM-co-TMSPMA-co-HPMA) copolymer films prepared at a final concentration of 10 mg·mL^−1^ decreased from 5.13 at 20 °C to approximately 1.68 at 37 °C, resulting in an excluded water content of 67.25 wt%. A comparable trend with respect to temperature was observed in the copolymer films prepared at the final concentrations of 50 and 100 mg·mL^−1^, albeit with lower swelling ratios. Specifically, at 20 °C, the swelling ratios were nearly 3.39 and 3.03, respectively, while at higher temperatures, they decreased to 1.41 and 1.33. Water discharge accounted for about 58.41 wt% and 56.11 wt%, respectively.

The weight reductions verified that the copolymer films displayed thermally responsive properties in three cases by phase transition of PNIPAM side chains. When immersed in deionized water at 20 °C, the copolymer films exhibited enhanced swelling due to the synergistic effect of hydrated hydrogen bonds formed between water molecules and hydrophilic amide bonds, hydroxyl groups, and siloxane bonds. Heating the copolymer films to 37 °C activated strong syneresis by enhancing hydrophobic interactions and non-hydrous hydrogen bonds within the copolymer chains, leading to the expulsion of excess water from the interpenetrating copolymer networks [15]. The findings above suggested that P(NIPAM-co-TMSPMA-co-HPMA) copolymers grafted onto the platinum electrode retained their temperature sensitivity. Furthermore, as the concentration of the preparation solution increased, the copolymer films exhibited a nonlinear reduction in their equilibrium swelling ratio at both 20 °C and 37 °C. This occurred because both the grafting density and crosslinking degree of the copolymer films increased progressively as the solution concentration rose, resulting in a reduction of the copolymer films’ water absorption capacities and subsequently, a decline in their equilibrium swelling ratios.

#### 2.2.4. Surface Wettability

**Figure 3:**
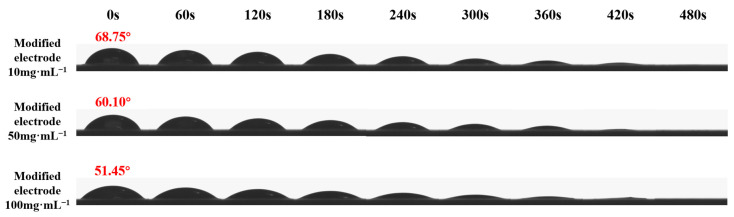
Contact angles of P(NIPAM-co-TMSPMA-co-HPMA) copolymer film-modified platinum working electrodes against preparation concentration and contact time.

The representative variations in static contact angle images for modified electrodes over time, ranging from 0 s to 480 s, are displayed in Figure 3. The contact angle of bare electrodes was approximately 88.5°, indicating the slightly water-repellent surface of the platinum electrode.

The sensitivity of the contact angles of electrodes modified with P(NIPAM-co-TMSPMA-co-HPMA) copolymer films was observed towards both preparation concentration and contact time. As the preparation concentration increased, the contact angles of the modified electrodes gradually decreased. The elevated concentrations resulted in the formation of denser copolymer films on the surface of working electrodes, potentially due to decreases in surface roughness. Meanwhile, the increase of hydrophilic groups within the copolymer films also led to a decrease in surface contact angle [32,33]. Furthermore, a notable decrease in the contact angles of the modified electrodes occurred as time progressed. As water droplets gradually penetrated into the copolymer films, they diffused and spread across the material surfaces. This phenomenon demonstrated that the infiltration of water into P(NIPAM-co-TMSPMA-co-HPMA) copolymer films was a dynamic process that was influenced by material composition and structure.

#### 2.2.5. Surface Morphology

**Figure 4:**
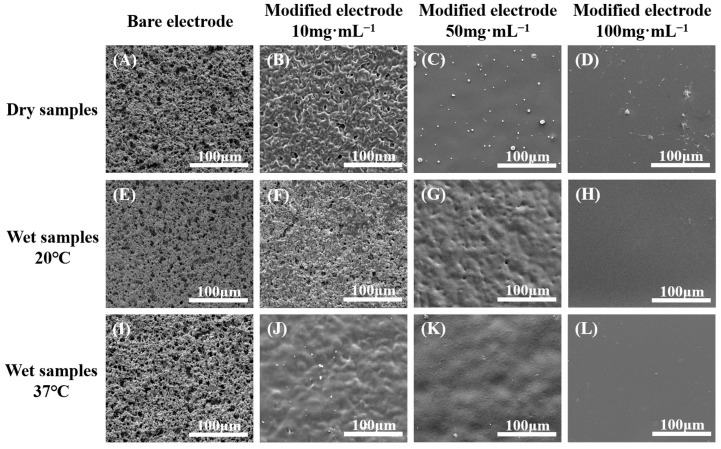
SEM images of bare working platinum electrodes and P(NIPAM-co-TMSPMA-co-HPMA) copolymer film-modified platinum electrodes, including dry states (**A**–**D**) and appearances after being soaked in deionized water at 20 °C (**E**–**H**) and 37 °C (**I**–**L**) for 3 h, followed by drying in a vacuum oven at their respective operating temperatures.

Figure 4 illustrates various representative surface topographies of bare electrodes and those modified with copolymer films under different conditions. The bare SPPEs exhibited rough and porous surfaces composed of electrically conductive platinum nanoparticles at both 20 °C and 37 °C. The modified working electrodes displayed significant alterations in surface morphology, marked by substantial filling of the surface pores. The surface morphology of the modified platinum electrodes varied depending on the preparation concentration and water temperature. When comparing the SEM images of the samples without immersion in deionized water, it was observed that the surface microstructure of P(NIPAM-co-TMSPMA-co-HPMA) copolymer films prepared at a final concentration of 10 mg·mL^−1^ exhibited a porous and rough appearance. In contrast, the surfaces of the copolymer films formed at higher final concentrations of 50 and 100 mg·mL^−1^ appeared to be more dense and smoother.

Upon immersion in water at various applied temperatures, the surface structure of the modified platinum electrodes underwent corresponding changes in accordance with water temperatures. Notably, the surface configuration of the film prepared using a 10 mg·mL^−1^ copolymer solution exhibited the most significant alterations with changes in temperature. As the temperature rose above the LCST, the surface transformed from a porous, rough, and loose texture to a non-porous, smooth, and dense structure. The film prepared with a 50 mg·mL^−1^ copolymer solution displayed a distinct wavy shape accompanied by a specific pore structure at 20 °C. However, at 37 °C, the waves smoothed out, and the pores largely disappeared. Conversely, the surface structure of the films prepared with a 100 mg·mL^−1^ solution were indistinct in SEM images across different temperatures, appearing generally smooth in both instances.

The SEM findings confirmed the robust attachment of the copolymer films to SPPEs, demonstrating no signs of delamination even under varying temperature conditions. This likely resulted from the covalent tethering, which was a crucial factor for practical applications [16,18]. Furthermore, the diverse surface morphologies observed with temperature changes suggested that the copolymer films maintained the temperature-responsive behavior exhibited by copolymers in solution.

#### 2.2.6. Amperometric Response

**Figure 5:**
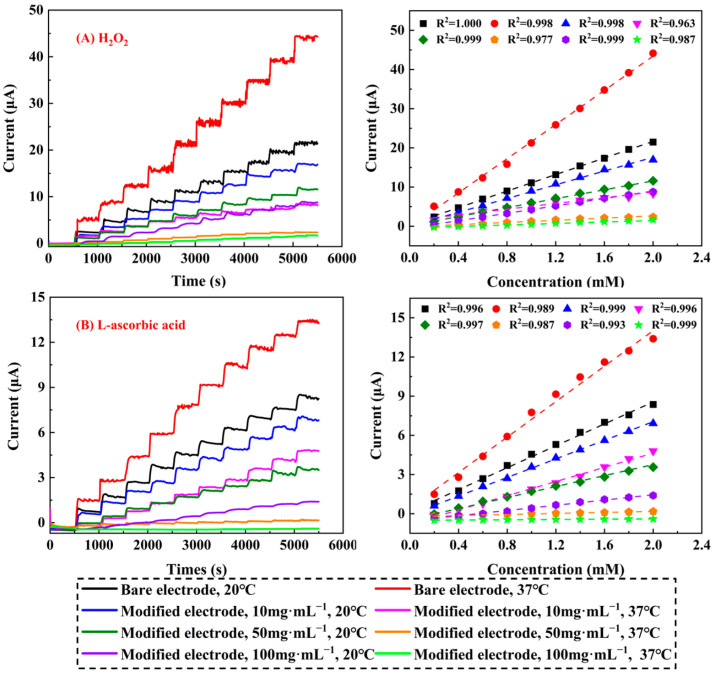
Amperometric responses and corresponding calibration curves of sensors with bare SPPEs and bearing different P(NIPAM-co-TMSPMA-co-HPMA) copolymer films under sequential additions of H_2_O_2_ (**A**) and L-ascorbic acid (**B**) with the concentration of 0.2 mM in PBS solution (pH = 7.4) at the working potential of 0.65 V and the applied temperature of 20 °C and 37 °C.

Figure 5 showed the amperometric current–time responses to H_2_O_2_ (A) and L-ascorbic acid (B) during consecutive additions of these analytes to phosphate-buffered solution (PBS). In the case of bare electrodes, the electrocatalytic responses towards H_2_O_2_ and L-ascorbic acid were observed to be more intense and rapid at 37 °C compared to 20 °C, and the amperometric responses to H_2_O_2_ and L-ascorbic acid at 37 °C were approximately 1.945 times and 1.676 times greater than those observed at 20 °C, respectively. This was a typical observation, since the increased temperatures tended to expedite the decomposition of H_2_O_2_ and L-ascorbic acid.

Under identical test conditions, the bare electrodes exhibited larger and more rapid responses than the modified electrodes. Notably, all modified biosensors reached 95% of their maximum steady-state responses to H_2_O_2_ and L-ascorbic acid within 350 s. This underscored the favorable electrocatalytic activity of P(NIPAM-co-TMSPMA-co-HPMA) copolymer films towards electrolytes. At both 20 °C and 37 °C, the three types of modified electrodes exhibited distinct and intriguing differences in their electrocatalytic activity. It was noteworthy that the signal strengths exhibited by these modified electrodes were inversely opposite to those of bare electrodes at both temperatures. Regardless of the preparation concentrations, P(NIPAM-co-TMSPMA-co-HPMA) copolymer film-modified electrodes displayed enhanced amperometric responses when catalyzing H_2_O_2_ and L-ascorbic acid at 20 °C. The alterations in the electrocatalytic activity of the electrodes modified with these films above and below the LCST were attributed to the temperature-dependent phase transition characteristics of the resulting copolymer films. Upon exposure to temperature stimuli ranging from 20 °C to 37 °C, all the copolymer films experienced hydration/dehydration transitions, resulting in the expansion/contraction of the polymeric films [34,35,36]. This caused two distinct states of electrocatalytic activity in the modified electrodes when reacting with electrolytes. However, as the preparation concentration increased, the surface morphologies of the modified working electrodes underwent significant changes, as evident from the SEM results. Consequently, the thermoresponsive characteristics of the modified sensors in relation to the temperature of the aqueous solution also showed corresponding alterations. Ascorbate response through P(NIPAM-co-TMSPMA-co-HPMA) copolymer films was about one third of that to H_2_O_2_ for bare and modified electrodes. Given the anionic nature of L-ascorbic acid, the additional Coulomb transport resistance seemed more likely to be the reason for the greater rejection of L-ascorbic acid [37]. The calibrations suggested a linearizing effect with the concentration of H_2_O_2_ or L-ascorbic acid.

When copolymer film-loading was increased in a sensor, the response magnitude and speed of H_2_O_2_ or L-ascorbic acid both also decreased, which could be attributed to the denser film barriers. The amperometric response was considered to be only diffusion controlled, and a preliminary estimation of the diffusion coefficient (*D*) could be derived using the formula *D* = 0.1388*L*^2^/*t*_0.5_, as previously stated [37,38]. This simplified and precise relationship stemmed from two universal equations, which comprehensively depicted the amperometric electrode’s transient output, ranging from its initial activation to its equilibrium response. For this purpose, it was necessary to compute the concentration profiles of film solutes. These profiles were obtained by employing the formulas *T* = *Dt*/*L*^2^ and *X* = *x*/*L*, where *T* represented the dimensionless time and *x* signified the spatial coordinate within the film. Additionally, the multiplier of 0.1388 originated from the fully simulated profile corresponding to the dimensionless time. It can be seen from Table 2 that the diffusion coefficients of the copolymer films at 20 °C were significantly higher than those at 37 °C under the same preparation concentration. This was because the surfaces of the copolymer films became denser due to dehydration and contraction at 37 °C, resulting in a reduction in film thickness. As a result, the electrolytes took longer to pass through the film layer and contact the platinum nanoparticles, leading to smaller diffusion coefficients. In addition, the diffusion coefficients exhibited clear downward trends with the increase in the preparation concentration under the same temperature, which was consistent with the expected results.

#### 2.2.7. Cyclic Voltammograms

**Figure 6:**
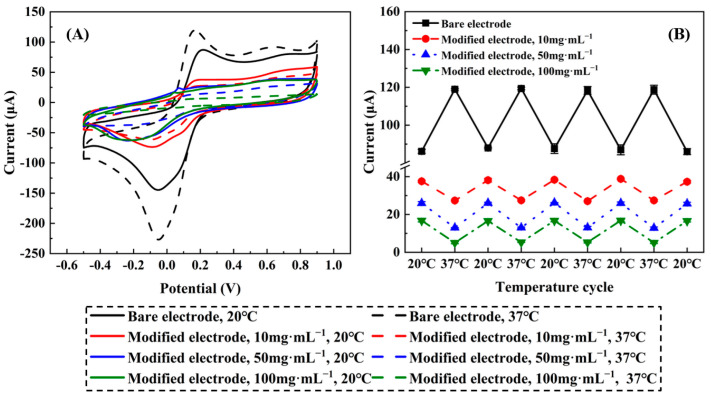
CV responses (**A**) and variation of peak currents (**B**) of sensors with bare SPPEs and bearing different P(NIPAM-co-TMSPMA-co-HPMA) copolymer films in 0.1 M PBS solution (pH = 7.4) containing 0.1 M KCl and 5 mM K_3_[FeCN_6_] at working potentials from −0.5 V to 0.9 V and applied temperatures of 20 °C and 37 °C.

Figure 6 displays the CV curves of bare and modified platinum electrodes at 20 °C and 37 °C. In the group of bare electrodes, the CV curve at 37 °C was significantly elevated compared to that at 20 °C, with the current peak of the former nearly 1.381 times as large as that of the latter. The reason for this was that the increased temperature enhanced the redox reaction of K_3_[Fe(CN)_6_]. However, a marked difference was observed in the CV curves of the modified electrodes. The CV curves at 37 °C were clearly lower than those at 20 °C, with the ratio of current peaks between the former and the latter being approximately 0.729, 0.500, 0.301, corresponding to the preparation concentrations of 10, 50, and 100 mg·mL^−1^, respectively. This was because P(NIPAM-co-TMSPMA-co-HPMA) copolymer films became shrunken and dense at 37 °C, leading to weakened electrical signals, whereas the films swelled and became loose at 20 °C, thus enhancing their responses. A vertical comparison of experimental results at the same temperature but with different concentrations revealed that as the preparation concentration increased, the current peak values gradually decreased. This was a natural consequence of the increased thickness of the films covering the platinum electrode surfaces.

When both bare and modified SPPEs were immersed in a PBS buffer solution and repeatedly subjected to temperature alternations, the CV peak currents exhibited a reversible trend that manifested as distinct “M” or “W”-shaped patterns. The values of peak currents returned exactly to the ones recorded during the initial measurements at the corresponding temperatures for all four thermal cycling steps, indicating the absence of hysteresis or drift. These findings underscored the copolymer films’ cycling stability and their suitability for applications involving temperature variations.

The electrochemical active surface area (EASA) of both bare and modified platinum electrodes could be approximated by analyzing the hydrogen adsorption/desorption peaks observed in the CV curves. Given a hydrogen monolayer adsorption charge of $Q_{H}^{0}$ = 0.21 mC∙cm^−2^, the EASA could be determined through the following calculation [39,40]:

$$EAS=\frac{Q_{H}}{Q_{H}^{0}}$$

where *Q_H_* is the integrated charge derived from the hydrogen adsorption/desorption peak areas in the CV curves.

Table 3 presents the results of EASA measurements for bare and modified platinum electrodes across various temperatures. For bare electrodes, the increase in the peak area observed in the CV curve at 37 °C indicated that the EASA was significantly higher compared to the results at 20 °C. This phenomenon could be attributed to the temperature effects discussed earlier. As the temperature rose from 20 °C to 37 °C, the rate of electrochemical reactions generally increased due to the higher kinetic energy of the reactants. This acceleration in reaction kinetics allowed for a greater utilization of the electrode surface, resulting in an apparent increase in EASA. Consequently, the CV curve at 37 °C exhibited a larger peak area, which was proportional to the number of active electrochemical sites accessible for charge transfer reactions. Therefore, it was reasonable to expect the EASA values calculated from CV measurements to be significantly higher at 37 °C compared to 20 °C, reflecting the increased electrochemical reactivity of the electrode surfaces at the higher temperature.

Bare SPPEs possessed a clear advantage in terms of EASA, demonstrating significantly higher values compared to the modified platinum electrodes at the same temperature. The presence of the copolymer film covering the SPPEs effectively reduced the exposed surface area of the platinum electrodes, thereby leading to a decrease in EASA. Nevertheless, K_3_[Fe(CN)_6_] was capable of penetrating the intact mesopores of the films to reach the electrode surfaces, resulting in acceptable EASA values for the modified electrodes. A lower EASA inevitably translates to smaller exposed surface areas of the platinum electrodes. This implied that an increase in grafting density and film thickness or a decrease in pore size and pore number were associated with reduced EASA of P(NIPAM-co-TMSPMA-co-HPMA) copolymer films, and vice versa.

#### 2.2.8. Stability, Repeatability and Selectivity

Despite some notable advances in science, thermoresponsive sensors still necessitate significant enhancements in material choices and device configurations to ensure long-lasting stability, reproducibility, and selectivity. These attributes are vital for sensors to function effectively in environments beyond the sheltered confines of a laboratory [41,42]. Consequently, the stability, including both the operational and storage stability, as well as the repeatability, whether in a single sensor or multiple parallel sensors, of P(NIPAM-co-TMSPMA-co-HPMA) copolymer film-modified SPPEs was thoroughly evaluated.

To assess the stability, 10 rounds of CV scans for each group of modified platinum electrodes within a potential range of −0.5 V to +0.9 V were conducted. These scans were carried out in a PBS buffer that contained 0.1 M KCl and 5 mM K_3_[Fe(CN)_6_], with a scan rate of 100 mV∙s^−1^ and at temperatures of 20 °C and 37 °C. The results indicated that the peak currents observed in the cyclic voltammograms remained largely unchanged. Furthermore, after sealed storage of the various copolymer film-modified platinum electrodes in a refrigerator at 4 °C for four weeks, their responses to 0.2 mM H_2_O_2_ were measured. Notably, the electrocatalytic responses maintained approximately 94.2 ± 3.8% of their initial currents. Additionally, the different modified platinum electrodes were immersed in a PBS solution with a pH of 7.4, at both 20 °C and 37 °C, for a duration of four weeks each. During this period, the amperometric responses to 0.2 mM H_2_O_2_ were also monitored. The results revealed that the electrocatalytic signals retained 92.5 ± 2.6% and 91.7 ± 1.9% of their initial values, respectively, indicating excellent stability.

To evaluate the repeatability, randomly selected copolymer film-modified SPPEs from each group were utilized to measure the amperometric responses to 0.2 mM H_2_O_2_ in parallel, both at 20 °C and 37 °C, for a total of 10 times. The results indicated that the relative standard deviation (RSD) of the response current was 3.4 ± 0.7% and 2.8 ± 0.6%, respectively, at the two temperatures. Furthermore, three parallel modified platinum electrodes from each group were electrochemically assayed with 0.2 mM H_2_O_2_, both at 20 °C and 37 °C. By comparing their amperometric responses, the RSD was 2.4 ± 0.3% and 2.1 ± 0.4%, respectively. These findings clearly demonstrated that P(NIPAM-co-TMSPMA-co-HPMA) copolymer film-modified SPPEs exhibited excellent repeatability with a relatively high degree of accuracy.

The selectivity remained unchanged between the bare and modified platinum electrodes. The surfaces of the SPPEs were coated and interconnected by P(NIPAM-co-TMSPMA-co-HPMA) films, which possessed mesoporous opening structures. Variations in temperature led to sudden alterations in the pore sizes and pore numbers of the copolymer films. However, these changes solely impacted the intensity of electrical signals and did not alter the selectivity of the sensors. Larger pores or an increased number of pores within the copolymer films facilitated the rapid and extensive arrival of electrolytes to the electrode surfaces, thereby amplifying the electrical responses. Conversely, a decrease in pore size and count hindered the diffusion of electrolytes to the platinum electrode surfaces, resulting in a weakening of the electrical signal. Notably, the diffusion rate of the electrolytes became the pivotal factor influencing the overall process rate, owing to the interference caused by the copolymer films, outweighing the redox reaction rate.

## 3. Materials and Methods

### 3.1. Materials

P(N-isopropylacrylamide-co-3-trimethoxysilylpropyl methacrylate-co-hydroxypropyl methacrylate), P(NIPAM-co-TMSPMA-co-HPMA) copolymers were synthesized by free radical polymerization with a molar ratio of monomers of 20:1:1 according to our investigations [22]. H_2_O_2_, L-ascorbic acid, KCl, K_3_[Fe(CN)_6_], and PBS were provided by Sigma-Aldrich (St. Louis, MO, USA). SPPEs (DS550) were obtained from DropSens (Asturias, Spain).

### 3.2. Preparation of P(NIPAM-co-TMSPMA-co-HPMA) Copolymer Film Modified Platinum Working Electrode

P(NIPAM-co-TMSPMA-co-HPMA) copolymers were initially dissolved in absolute ethanol with the final concentrations of 10, 50, and 100 mg∙mL^−1^. These solutions were then agitated at 20 °C for 12 h. Subsequently, all copolymer solutions underwent filtration using Millipore filters with a pore size of 0.2 μm. Finally, 10 μL aliquots of the various copolymer solutions were deposited onto the platinum working electrode surfaces of the SPPEs. After drying in air for 30 min at 20 °C, the coatings were subjected to annealing for 3 h at 125 °C under a vacuum to promote the crosslinking of 3-trimethoxysilyl groups and their reaction with hydroxyl groups. To eliminate any unreacted copolymers, the modified SPPEs were immersed and subsequently rinsed thoroughly with deionized water, followed by vacuum drying at 20 °C.

### 3.3. Characterization of P(NIPAM-co-TMSPMA-co-HPMA) Copolymer Film-Modified Platinum Working Electrodes

#### 3.3.1. Chemical Composition

The functional groups of P(NIPAM-co-TMSPMA-co-HPMA) copolymer film-modified platinum electrodes were analyzed through ATR-FTIR spectroscopy employing a Bruker TENSOR27 spectrometer (BRUKER OPTIK GmbH, Ettlingen, Germany) within the wavenumber range of 4000 to 500 cm^−1^.

#### 3.3.2. Grafting Density

The grafting density of P(NIPAM-co-TMSPMA-co-HPMA) copolymer film-modified SPPEs was quantitatively determined through gravimetric measurements. This calculation was based on the mass difference observed before and after the functionalization process, normalized by the outer surface area of the platinum working electrode.

$$d_{g}=\frac{W_{f}-W_{0}}{A}$$

where *d_g_* was grafting density, *W*_0_ represented the initial dry weight of the unmodified SPPEs, and *W_f_* denoted the final dry weight of the modified SPPEs after undergoing the coating, annealing, washing, and drying procedures. The outer surface area of the platinum working electrode with a diameter of 4 mm, was designated as *A*. This metric for grafting density has been widely used by researchers as a proxy for the extent of grafting achieved [43,44]. To evaluate the grafting density for each type of copolymer, six samples were utilized.

#### 3.3.3. Equilibrium Swelling

For equilibrium swelling measurements, the modified SPPEs were initially dried in a vacuum oven at 20 °C for 12 h. Subsequently, they were weighed using a Semi-Micro Lab Balance (Quintix35-1CN, Sartorius Lab Instruments GMBH & Co. KG, Goettingen, Germany) with a measurement accuracy of 0.01 mg (*W*_*dry*_). Following this, both bare and modified electrodes were immersed and maintained in a 0.1 M PBS solution (pH 7.4) at either 20 °C or 37 °C for a minimum duration of 3 h. We removed the electrodes from the PBS solution, wiped off the surface moisture, and immediately measured the weights (*W*_*wet*,20°C_, *W*_*wet*,37°C_). The equilibrium swelling ratio (ESR) was defined as [45,46]:

$$\mathrm{ESR}=\frac{W_{wet}-W_{dry}}{W_{f}-W_{0}}$$

#### 3.3.4. Surface Wettability

To conduct contact angle measurements, the SPPEs coated with the P(NIPAM-co-TMSPMA-co-HPMA) copolymer film were first dried in a vacuum oven at 20 °C. They were then meticulously placed on the operating platform of the Drop Shape Analyzer (DSA100, KRüSS GmbH, Hamburg, Germany). For these measurements, bare platinum electrodes served as reference substrates for comparison. At 20 °C, static contact angles (designated as *θ*) were measured for 2 µL sessile droplets of deionized water on the surfaces. Each sample underwent six separate trials. Immediately upon deposition of the droplet onto the surface, the associated images and contact angle values were captured and collected every 60 s thereafter until 480 s. Subsequently, these data were processed using Drop Shape Analysis Software v1.92.1.1 for comprehensive analysis.

#### 3.3.5. Surface Morphology

Both bare and P(NIPAM-co-TMSPMA-co-HPMA) copolymer film-modified SPPEs underwent gold sputtering using an automatic sputter coating machine (Agar Scientific Ltd., Stansted, UK). Furthermore, the modified electrodes were immersed in deionized water for 3 h, specifically at either 20 °C (below the LCST) or 37 °C (above the LCST). Subsequently, they were vacuum dried at the respective temperatures before being coated with gold. Under standard vacuum conditions at an acceleration voltage of 10 kV, the Quanta 400 FEG instrument (FEI, Hillsboro, OR, USA) was utilized to observe the surface morphology of the specimens via scanning electron microscopy (SEM).

#### 3.3.6. Amperometric Response

Amperometric measurements were conducted employing a benchtop Electrochemical Analyzer (CHI842C, CH Instruments Inc., Austin, TX, USA) and a three-electrode setup, comprising a platinum working electrode coated with a P(NIPAM-co-TMSPMA-co-HPMA) copolymer film, a platinum high-temperature curing ink counter electrode, and a silver reference electrode. PBS with a concentration of 0.1 M and a pH of 7.4 was utilized for all electrochemical measurements. Current–time (*i*-*t*) curves were obtained by conducting measurements in a stirred vessel, where 40 μL aliquots of 0.1 mM H_2_O_2_ or L-ascorbic acid were successively added to a 20 mL PBS solution, resulting in a stepwise increase of 0.2 mM. These measurements were performed at an operating potential of 0.65 V and under either 20 °C (below the LCST) or 37 °C (above the LCST) temperature conditions.

A preliminary estimation of the diffusion coefficient (*D*) could be calculated as [37,38]:

$$D=0.1388L^{2}/t_{0.5}$$

where *L* was film thickness, and *t*_0_._5_ was the measured half-time value of dynamic amperometric responses of H_2_O_2_ with independent P(NIPAM-co-TMSPMA-co-HPMA) copolymer film barrier. Film thickness was determined via a micrometer screw gauge (0~25 mm, Draper, Chandler’s Ford, UK).

#### 3.3.7. Cyclic Voltammograms

CV curves of bare and modified SPPEs were obtained by scanning the potential from −0.5 V to +0.9 V in a PBS buffer containing 0.1 M KCl and 5 mM K_3_[Fe(CN)_6_] at a rate of 100 mV∙s^−1^ while maintaining a temperature of 20 °C and 37 °C, respectively. Variations in the CV peak currents were recorded at 20 °C and 37 °C, and this process was repeated four times to monitor for any changes in the CV peak current.

## 4. Conclusions

This study has delved into the enhancement of temperature-sensitive sensor performance through modification with PNIPAM, exploring its tailored thermoresponsiveness. P(NIPAM-co-TMSPMA-co-HPMA) copolymers were successfully grafted onto the surfaces of platinum working electrodes of SPPEs using a two-step film formation method in this study. With an increase in the preparation concentration, the grafting densities and film thicknesses of the copolymers on the platinum electrodes increased nonlinearly. However, the equilibrium swelling ratios, surface contact angles, diffusion coefficients, and electrochemical active surface areas of the copolymer films on the platinum electrodes exhibited downward trends at the temperatures above and/or below the LCST. Additionally, when comparing any given preparation concentrations, the measured values at 20 °C were significantly higher than those at 37 °C, indirectly confirming the temperature-sensitive characteristics of the copolymer films used to modify the platinum electrodes. The surfaces of the modified electrodes were rougher and more porous at 20 °C than at 37 °C, directly revealing the temperature-sensitive behavior of the copolymer films. The amperometric current–time responses highlighted the excellent electrocatalytic activity of the P(NIPAM-co-TMSPMA-co-HPMA) copolymer films towards electrolytes. Regardless of the preparation concentration, the electrodes modified with P(NIPAM-co-TMSPMA-co-HPMA) copolymer films exhibited enhanced amperometric responses when catalyzing H_2_O_2_ and L-ascorbic acid at 20 °C compared to 37 °C. The comparison of cyclic voltammetry results obtained at the same temperature but with varying concentrations revealed a gradual decrease in current peak values as the preparation concentrations increased. However, upon repeated temperature changes, reversibility of CV peaks was observed, demonstrating the stability and durability of the temperature-sensitive electrochemical sensors. In summary, this study has successfully enhanced the performance of electrochemical sensors through the introduction and customized design of PNIPAM. This not only expands the application domain of temperature-responsive polymers, but also paves a new path for the further development and optimization of sensors.

## Figures and Tables

**Table 1:** Grafting densities and equilibrium swelling ratios of P(NIPAM-co-TMSPMA-co-HPMA) copolymer film-modified platinum working electrodes.

Sample	*W*_f_—*W*_0_ (mg)	*W*_wet,20°C_—*W*_dry,20°C_ (mg)	*W*_wet,37°C_—*W*_dry,37°C_ (mg)	*d*_g_ (mg·cm^−2^)	ESR_20°C_	ESR_37°C_
Modified electrode, 10 mg·mL^−1^	0.31 ± 0.02	1.59 ± 0.07	0.52 ± 0.11	2.53 ± 0.16	5.13 ± 0.23	1.68 ± 0.35
Modified electrode, 50 mg·mL^−1^	0.51 ± 0.03	1.73 ± 0.13	0.72 ± 0.08	4.16 ± 0.24	3.39 ± 0.25	1.41 ± 0.16
Modified electrode, 100 mg·mL^−1^	0.73 ± 0.08	2.21 ± 0.16	0.97 ± 0.10	5.95 ± 0.65	3.03 ± 0.22	1.33 ± 0.14

Note: *W*_0_—initial dry weight of unmodified SPPEs; *W*_f_—final dry weight of modified SPPEs after coating, annealing, washing, and drying procedures; *W*_dry_—dry weight of modified SPPEs; *W*_wet_—wet weight of modified SPPEs; *d*_g_, grafting density; ESR—equilibrium swelling ratio.

**Table 2:** Diffusion coefficients (*D*) of modified platinum electrodes.

Sample	*L*_20°C_ (cm)	*L*_37°C_ (cm)	*t*_0_._5,20°C_ (s)	*t*_0_._5,37°C_ (s)	*D*_20°C_ (cm^2^·s^−1^)	*D*_37°C_ (cm^2^·s^−1^)
Modified electrode, 10 mg·mL^−1^	0.0017	0.0010	12.67	23.40	3.166 × 10^−8^	5.932 × 10^−9^
Modified electrode, 50 mg·mL^−1^	0.0035	0.0018	68.18	90.50	2.494 × 10^−8^	4.969 × 10^−9^
Modified electrode, 100 mg·mL^−1^	0.0047	0.0022	145.24	164.47	2.111 × 10^−8^	4.085 × 10^−9^

**Table 3:** Electrochemical active surface areas (EASAs) of bare and modified platinum electrodes.

Sample	EASA_20°C_ (m^2^·g^−1^)	EASA_37°C_ (m^2^·g^−1^)
Bare electrode	117.78 ± 4.76	162.66 ± 5.66
Modified electrode, 10 mg·mL^−1^	64.26 ± 2.48	46.79 ± 0.95
Modified electrode, 50 mg·mL^−1^	44.33 ± 1.49	28.68 ± 1.23
Modified electrode, 100 mg·mL^−1^	22.17 ± 0.19	8.64 ± 0.86

## Data Availability

All data are contained within the article.
